# Scalp angiosarcoma treated with linear accelerator-based boron neutron capture therapy: A report of two patients

**DOI:** 10.1016/j.ctro.2022.02.006

**Published:** 2022-02-18

**Authors:** Hiroshi Igaki, Naoya Murakami, Satoshi Nakamura, Naoya Yamazaki, Tairo Kashihara, Akira Takahashi, Kenjiro Namikawa, Mihiro Takemori, Hiroyuki Okamoto, Kotaro Iijima, Takahito Chiba, Hiroki Nakayama, Ayaka Takahashi, Tomoya Kaneda, Kana Takahashi, Koji Inaba, Kae Okuma, Yuko Nakayama, Kazuaki Shimada, Hitoshi Nakagama, Jun Itami

**Affiliations:** a Department of Radiation Oncology, National Cancer Center Hospital, Tsukiji 5-1-1, Chuo-ku, Tokyo 104-0045, Japan; b Division of Research and Development for Boron Neutron Capture Therapy, National Cancer Center Exploratory Oncology Research & Clinical Trial Center, Tsukiji 5-1-1 Chuo-ku, Tokyo 104-0045, Japan; c Department of Medical Physics, National Cancer Center Hospital, Tsukiji 5-1-1 Chuo-ku, Tokyo 104-0045, Japan; d Department of Dermatologic Oncology, National Cancer Center Hospital, Tsukiji 5-1-1 Chuo-ku, Tokyo 104-0045, Japan; e Department of Radiological Science, Graduate School of Human Health Sciences, Higashi-ogu 7-2-10 Arakawa-Ku, Tokyo 116-8551, Japan; f National Cancer Center Hospital, Tsukiji 5-1-1 Chuo-ku, Tokyo 104-0045, Japan

**Keywords:** Boron neutron capture therapy (BNCT), Linear accelerator-based BNCT system, Solid-state Li target, First-in-human study, Scalp-angiosarcoma

## Abstract

**Purpose:**

This study reports the first-in-human use of a linear accelerator-based boron neutron capture therapy (BNCT) system and the first treatment of patients with scalp-angiosarcoma with accelerator-based BNCT.

**Patients and methods:**

A single-center open-label phase I clinical trial has been conducted using the system since November 2019. Patients with a localized node-negative scalp-angiosarcoma along with the largest diameter of the tumor ≤ 15 cm who refused primary surgery and chemotherapy were enrolled. After administration of boronophenylalanine (BPA), a single treatment of BNCT with the maximum dose delivered to the skin being 12 Gy-Eq was performed. The safety and effectiveness of accelerator-based BNCT therapy for localized scalp angiosarcoma were evaluated.

**Results:**

Scalp-angiosarcoma of the two patients did not develop the dose-limiting toxicity in the clinical trial. They reached CR within half a year and did not exhibit in-field failure 20 months after BNCT without any severe treatment-related adverse events. Although a grade 3 adverse event of an asymptomatic but increased serum amylase level was noted in both patients, it relieved without any treatment. Additionally, no severe acute dermatitis was observed for both patients, which is typically seen with conventional primary radiotherapy.

**Conclusions:**

It was suggested that BNCT would be a promising treatment modality for scalp-angiosarcoma, which is difficult to treat.

## Highlights

• Scalp-angiosarcoma is difficult to manage, and surgery is the primary therapy.
• BNCT can deliver highly effective particles more selectively to tumor cells.
• BNCT requires a high neutron flux that typically can only be supplied by a nuclear reactor.
• A linear accelerator-based BNCT system that can be installed in a hospital has been developed.
• Two scalp-angiosarcoma patients were effectively treated by the linear accelerator-based BNCT.

## Introduction

Boron neutron capture therapy (BNCT) utilizing an accelerator-based neutron source is a recently introduced in radiotherapy that is primarily based on ^10^B(n, α)^7^Li reactions. As the α and ^7^Li particles released during BNCT exhibit high linear energy transfer, the relative biological effectiveness of BNCT is greater than that of conventional radiotherapies [1]. As the range of the range of α and ^7^Li particles is comparable to the size of the target cells, if the concentration of ^10^B particles is several times higher in the tumor than in the surrounding tissues, it is possible to achieve a highly selective tumor cell killing. The high biological effectiveness via boron neutron capture reaction has been experimentally demonstrated [1].

Boronophenylalanine (BPA) is a widely used ^10^B-containing drug that is taken up by tumor cells via the L-type amino acid transporter 1 (LAT-1) [2], which is expressed at high levels by many tumors [3]. High LAT-1 expression is also associated with an unfavorable prognosis [4], so BNCT may be particularly beneficial in these tumors.

Clinical studies using BNCT have been applied in clinical setting to a number of malignacies, such as brain tumors, malignant melanoma, head and neck cancer, etc…, with favorable clinical outcomes [2], [5], [6]. However, the widespread use of BNCT in clinical medicine was limited by the regulatory and safety issues that are inherent to the necessity of using a nuclear reactor to produce an adequate neutron supply. Thanks to recent work, accelerator-based neutron sources have been introduced for BNCT that can be installed in a hospital [6], [7]. Suitable neutron beams can be obtained with a linear accelerator and a cyclotron. An accelerator-based BNCT system consisting of a radiofrequency quadrupole linear coupled to a lithium target was installed at our institution, the National Cancer Center Hospital (NCCH) in Tokyo, in December 2014, and has been available for clinical use since 2019 [7]. A phase I clinical trial has been conducted using this system for treating localized cutaneous malignant melanoma and scalp angiosarcoma since November 2019. The phase I clinical trial seeks to test the safety of the linear accelerator-based BNCT system, which adopts a vertical epithermal neutron beam, and to identify the maximum tolerable dose to the skin in BNCT [8]. As neutrons have a limited penetration depth, superficial tumors such as skin malignancies are deemed good candidates for BNCT. The detailed information for the penetration depth and CICS-1 can be found in our previous reports [9]. Of the various histopathologic types in skin malignancies, cutaneous malignant melanoma and angiosarcoma were selected because previous studies found that they highly express LAT-1 [10]. Both diseases most commonly affect the extremities and head and neck regions.

Surgery followed by postoperative radiation therapy and/or chemotherapy is the standard of care for scalp-angiosarcoma, an aggressive rare malignant tumor of vascular endothelial cells [11]. However, because scalp-angiosarcoma often affects elderly individuals with comorbidities and requires large resection (as tumors can have multiple foci, take up a large surface area, and have ill-defined borders), not all patients are candidates for radical resection. Conservative therapy with definitive radiation may therefore be a preferable for selecting or to select patients who may not tolerate the requisite reconstruction or who seek purely quality-of-life benefits. Primary radiation therapy using X-rays and/or electrons is an alternative treatment option. However, the local control rate of definitive radiation therapy ranges between 50% and 70%, which cannot be regarded as satisfactory [12], [13], [14], [15]. Severe radiation dermatitis is inevitable if a curative dose of photons or electrons is delivered to the skin [14], [15]. Primary surgery, therefore, remains the primary treatment option for scalp angiosarcoma [10].

This report describes the first-in-human application of a linear accelerator-based BNCT system to treat patients with scalp angiosarcoma.

## Materials and methods

### Clinical trial overview

The ongoing trial utilizes an investigational device (CICS-1, neutron irradiation device manufactured by Cancer Intelligence Care Systems, Inc.) and an investigational drug (SPM-011, ^10^B pharmaceutical provided by Stella Pharma Corporation). The trial is a single-center (NCCH), open-label study. The primary endpoint is to evaluate the incidence of dose-limiting toxicity at a predetermined radiation dose based on a 3 + 3 + 3 design, so at least 9 patients will be enrolled. Hence, the clinical trial is conducting as the dose escalation trial, and the level 1 dose was 12 Gy-Eq to skin. The level 1 dose was set as considering enough safe to perform BNCT based on previous reports about BNCT from the reactor [16], [17]. Blood test and physical examination evaluation for acute toxicities were performed 1, 3, 7, 30, 60, and 90 days after irradiation. Severity of adverse events were assessed using CTCAE v 5.0. Dose-limiting toxicity was defined as any treatment-related grade 4 or higher and serious adverse event within 90 days after irradiation. The duration of the clinical trial was up to 180 days, and the patients were followed up after the end of the clinical trial period. Patients diagnosed histopathologically with a cutaneous malignant melanoma or angiosarcoma without lymph node or distant metastasis can be enrolled in the trial. ^18^Fuluoro-BPA positron emission tomography is not performed in this trial while it is considered as one of the useful indicators of adaptation for BNCT. The trial protocol is registered with *ClinicalTrials.gov* (NCT04293289) and *JapicCTI* (JapicCTI-195062). The clinical trial is being conducted according to the Declaration of Helsinki and Good Clinical Practices defined by the International Conference on Harmonization and in compliance with the registered protocol. The clinical trial was approved by the institutional review boards at NCCH. All patients provided written informed consent prior to intervention.

### Case presentations

**Fig. 1:**
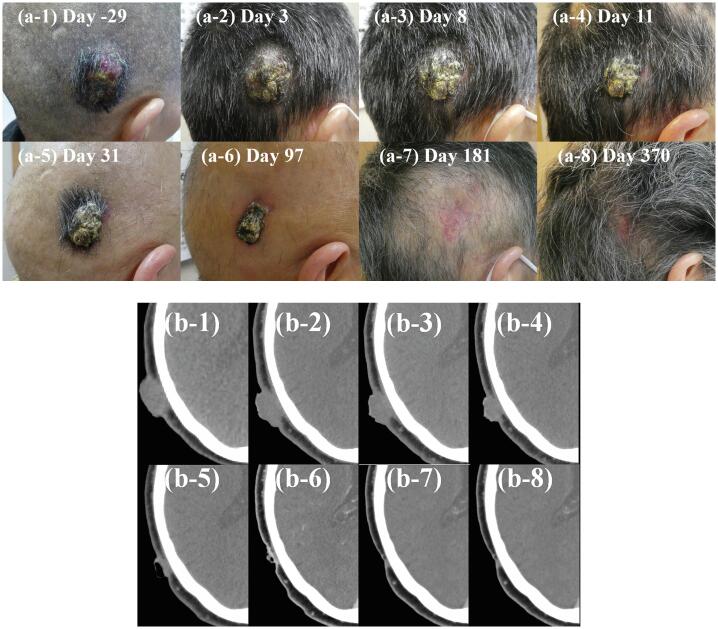
Time course of Patient 1 tumor before and after boron neutron capture therapy (BNCT). (a) Clinical photos and (b) Computed tomography scans obtained before treatment start (day −29) and on treatment days 3, 8, 11, 31, 97, 181, and 370. The patient achieved PR and CR on days 31 and 181, respectively, according to the RECIST 1.1 criteria.

Patient 1, a previously healthy 68-year-old female, was referred to us for the evaluation of a right temporodorsal scalp mass (Fig. 1). After standard diagnostic and staging studies, she was diagnosed with scalp angiosarcoma and had no lymph node nor distant metastasis. The tumor was 35 × 28 mm and 22 mm thick. She refused radical surgery followed by postoperative chemoradiotherapy due to its invasiveness and expected aesthetic outcomes, so she was referred for BNCT.

**Fig. 2:**
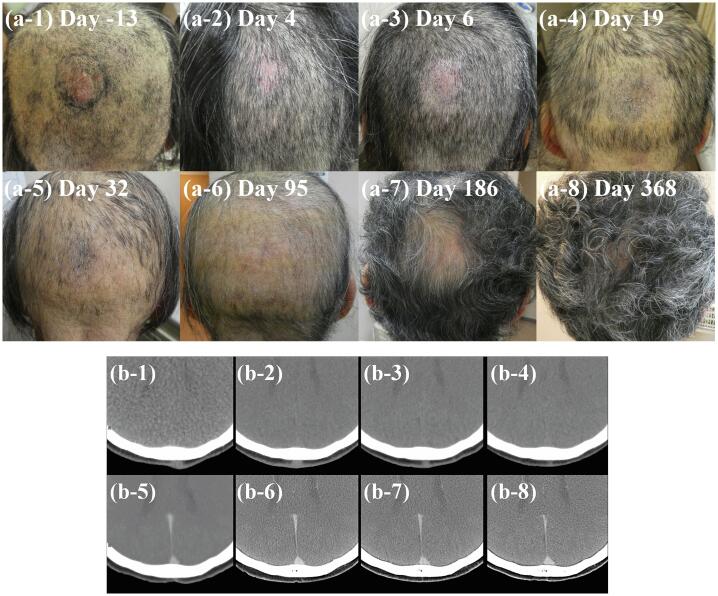
Time course changes in Patient 2 tumor before and after Boron Neutron Capture Therapy (BNCT). (a) Clinical photos and (b) computed tomography scans obtained before treatment start (day 13) and on treatment days 4, 6, 19, 32, 95, 186, and 368. The patient achieved PR and CR on days 4 and 19, respectively, according to the RECIST 1.1 criteria.

Patient 2, a previously healthy 66-year-old female was referred to us for evaluation of a dorsal scalp mass (Fig. 2). After standard diagnostic and staging studies, she was diagnosed with scalp angiosarcoma and had no lymph node nor distant metastasis. The tumor was 29 × 23 mm and 10 mm thick. This patient also refused radical surgery followed by postoperative chemoradiotherapy because of the same reason as patient 1, then, she was referred for BNCT.

### Treatment

Patients received continuous intravenous administration of 200 mg/kg/h of SPM-011 just before neutron irradiation for 2 h. After this 2-hour administration, the blood sampling was performed to measure the ^10^B concentration to blood using an inductivity coupled plasma optical emission spectrometer (SPS3500DD, Hitachi High-Tech Science Corporation, Tokyo, Japan). After them without a pause, continuous intravenous administration speed of SPM-011 was decreased to 100 mg/kg/h for the next 1 h [6]. Based on result of the ^10^B concentration measurement, the required proton charge was determined to deliver the maximum dose to skin of 12 Gy-Eq, and the neutron irradiation was then performed during the last 1 h administration. When the neutron irradiation to the patient was finished, the administration was stopped.

The distributions of neutron and gamma-ray in the patient body were calculated by Monte Carlo Simulation (Particle and Heavy Ion Transport code System (PHITS), ver. 3.020) [18]. Considering the elemental composition in each tissue, the calculated neutron distribution was multiplied by the kerma coefficient of boron, nitrogen, and hydrogen while the calculated gamma-ray distribution was multiplied by dose conversion factor [19], [20], [21]. As a result, the total physical BNCT dose, which composed of the boron, nitrogen, hydrogen, and gamma-ray doses, was derived. Additionally, in order to consider the compound biological effectiveness (CBE) in the boron dose and the relative biological effectiveness (RBE) in each dose component, the physical dose of each dose component is calculated by multiplying the corresponding CBE or RBE, and the total RBE-weighted dose was then calculated by taking the sum. It was noted that the boron concentration in the blood was assumed as 25 ppm on the treatment planning. Calculation parameters are summarized in Table 1 [6], [16], [17], [25]. According to Table 1, the boron concentration in the tumor was assumed as 87.5 ppm. In patient 1, the maximum and minimum expected doses to the gross tumor volume (GTV) were expected as 41.6 and 36.3 Gy-Eq, respectively, whereas those to the brain were expected as 5.3 and 0.5 Gy-Eq, respectively. Additionally, the maximum and minimum expected doses to the eyes were 2.4 and 0.7 Gy-Eq, respectively. The expected RBE-weighted dose to skin for patient 1 was 12 Gy-Eq, and its boron, nitrogen, hydrogen, and gamma-ray doses were then expected as 10.5, 0.5, 0.1, and 0.9 Gy-Eq, respectively. In patient 2, the maximum and minimum expected doses to the GTV were 41.3 and 30.5 Gy-Eq, respectively, whereas those to the brain were expected as 5.8 and 0.6 Gy-Eq, respectively. The maximum and minimum expected doses to the eyes were 1.4 and 0.7 Gy-Eq, respectively. The expected RBE-weighted dose to skin was also 12 Gy-Eq, and its boron, nitrogen, hydrogen, and gamma-ray doses were then expected as 10.4, 0.6, 0.1, and 0.9 Gy-Eq, respectively.

**Table 1:** Compound biological effectivenss, relative biological effectiveness, and boron concentration parameters in the treatment planning.

	CBE	RBE (Nitrogen)	RBE (Hydrogen)	RBE (gamma-ray)	Tissue-to-blood ratio of boron concentration
Tumor	4.0[6]	3.0[16]	3.0[25]	1.0	3.5[6]
Brain	1.34[6]	3.0[25]	3.0[25]	1.0	1.0[6]
Skin	2.5[6]	3.0[16]	3.0[25]	1.0	1.4[17]
CBE: Compound biological effectiveness. RBE: relative biological effectiveness.					

## Results

All data are current as of September 8, 2021.

### Treatment delivered

The neutron radiation dose was prescribed so that the maximum dose delivered to the skin was 12 Gy-Eq in both patients.

#### Patient 1

Patient 1 was the first treatment treated with neutron beam obtained with a linear accelerator coupled to a Li target. The measured boron concentration in the blood after the first 2-hour administration of SPM-011 was 42.2 ppm. Based on the measured boron concentration, the required proton charge and the treatment time was 9526 mC and 17 min. Deriving the neutron flux model reported in the previous studies, the epithermal neutron of 5.3 × 10^11^ cm^−2^ was provided by CICS-1 [7], [22].

#### Patient 2

The measured boron concentration in the blood after the first 2-hour administration of SPM-011 was 36.5 ppm. Based on the measured boron concentration, the required proton charge and the treatment time was 11,636 mC and 20 min. Deriving the neutron flux model, the epithermal neutron of 6.3 × 10^11^ cm^−2^ was provided by CICS-1.

### Clinical outcomes

#### Patient 1

**Fig. 3:**
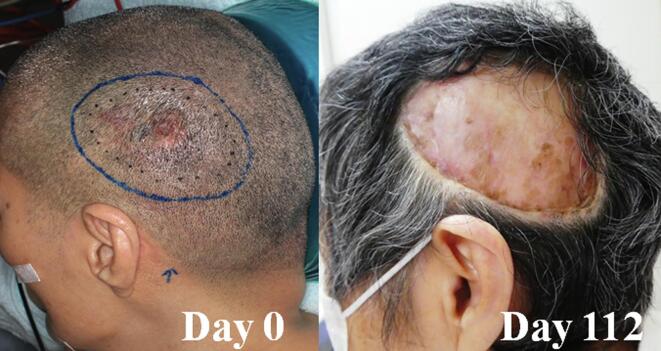
Time course of Patient 1′s extra-field relapse before and after local excision.

The patient’s tumor shrank slowly, reduced in thickness and reached PR and CR on days 31 and 181, respectively (Fig. 1). No disease in-field recurrence was noted over 21 months. It was noted that the maximum therapeutic field size was 15 cm in diameter on the clinical trial protocol (i.e., in-field), and the protocol was based on results of the several non-clinical trials in CICS-1. Grade 2 alopecia was noted on the treated skin on day 10, which improved to grade 1 on day 181. Table 2 summarizes radiation-induced toxicities, which all subsided spontaneously within a month. However, extra-field relapse occurred in the left parietal part of the head 15 months after BNCT. Because additional BNCT was not allowed by the protocol, salvage surgery was performed. A local excision with a 1-cm margin for the relapse area was performed 2 months later after the recurrence (7.5 × 10 cm) (Fig. 3).

**Table 2:** Treatment-related toxicities after boron neutron capture therapy in each patient.

	**Patient 1**		**Patient 2**	
	**Worst date (Grade)**	**Recovered date (Grade)**	**Worst date (Grade)**	**Recovered date (Grade)**
Hyperamylasemia	Day 1 (G3)	Day 8 (G0)	Day 1 (G3)	Day 32 (G0)
Pancreatic amylase/ salivary amylase [IU/L]	50/1798	59/71	26/2911	33/73
Alopecia	Day 10 (G2)	Day 181 (G1)	Day 19 (G2)	Day 186 (G0)
Nausea	Day 3 (G1)	Day 6 (G0)	Day 1 (G2)	Day 3 (G0)
Dermatitis	Day 15 (G1)	Day 31 (G0)	Day 6 (G1)	Day 19 (G0)
Fatigue	Day 2 (G1)	Day 6 (G0)	—	—
Neutropenia	Day 3 (G1)	Day 31 (G0)	—	—
Anemia	Day 3 (G1)	Day 31 (G0)	—	—
Lymphopenia	Day 3 (G2)	Day 8 (G0)	—	—
Leucopenia	Day 3 (G2)	Day 31 (G0)	—	—
Increased serum bilirubin	—	—	Day 1 (G1)	Day 6 (G0)

#### Patient 2

Fig. 2 shows the changes in the tumor over time. Patient 2 achieved PR and CR on days 4 and 19, respectively. No disease progression was noted during 20 months. Grade 2 alopecia on the treated skin was noted on day 19, which improved to grade 0 on day 186. Table 2 summarizes the radiation-induced toxicities sustained by this patient. Similar to Patient 1, these toxicities subsided spontaneously within 3 weeks.

### Specific adverse events

A grade 3 adverse event of an asymptomatic but increased serum amylase level that was more than five times greater than the upper limit of normal was noted in both patients. This event was observed the day after the BNCT doses were administered and recovered without therapy (patient 1: day 8, patient 2: day 32). Amylase isozyme analysis revealed that salivary amylase was responsible for this transient change (patient 1: total amylase 1848 IU/L, pancreatic amylase 50 IU/L, and salivary amylase 1798 IU/L; patient 2: total amylase 2937 IU/L, pancreatic amylase 26 IU/L, and salivary amylase 2911 IU/L). No other severe acute toxicities or delayed toxicities were observed in either patient.

## Discussion

Here we present the first report of patients with scalp angiosarcoma treated with an accelerator-based BNCT system. Both patients had safe outcomes and favorable short-term tumor responses.

While our follow-up period was relatively short, no in-field progression was noted in either of the two patients, though an extra-field local relapse was observed 15 months after BNCT in patient 1. Most local progression occurs within a year [14], so our preliminary results may be considered favorable. Neither patient went on to develop severe radiation dermatitis, presumably due to the high selectivity of BNCT to areas with a high boron concentration, as mentioned above. According to the previous reports, increased dose to the tumor is associated with improved of clinical outcome [15] while severe radiation dermatitis is inevitable [14], [15]. However, it is difficult for conventional photon or electron irradiation to increase the dose to the tumor while keeping skin dose within tolerable toxicities. Therefore, it could be possible that BNCT becomes a novel treatment option for cutaneous angiosarcoma in the future.

Alopecia was observed in both patients, approximately 3 weeks after BNCT. The expected threshold radiation doses for tissue reactions were reported in the ICRU publication 118, a comprehensive report on early and late effects of radiation in normal tissues and organs derived from conventional radiotherapy experience, with temporary and permanent alopecia being approximately 3 and 7 Gy in a single fraction, respectively, and alopecia being observed approximately three weeks after radiation exposure [23]. The maximum dose to the skin in our protocol was defined as 12 Gy-Eq, which was localized to the skin close to the tumor and was higher than the calculated threshold for permanent alopecia of 7 Gy-Eq in BNCT. Permanent alopecia was therefore observed in only very close areas of the scalp around the tumor, which were smaller than the skin areas over 7 Gy-Eq with an expected dimeter of 10–12 cm derived from our dose calculation. With respect to the post-treatment aesthetic outcomes, BNCT was clearly better than surgery (Fig. 1, Fig. 2).

The only grade 3 adverse event related to BNCT was transient asymptomatic hyperamylasemia, which was more than five-times greater than the upper limit of normal, and hence was categorized as a grade 3 adverse effect. The calculated mean ipsilateral and contralateral parotid gland doses for patient 1 were 4.3 and 1.2 Gy-Eq, respectively, and 2.0 and 1.9 Gy-Eq for patient 2, respectively. This adverse effect was noted in a previous report of BNCT used in the treatment of recurrent head and neck cancer, in which 86% of patients experienced hyperamylasemia [6]. According to previous reports, hyperamylasemia can occur after a dose to the parotid glands larger than 0.5–1 Gy delivered with the conventional radiotherapy [24]. It is therefore likely that radiation exposure to the salivary glands was the cause of these amylase levels. Fortunately, the hyperamylasemia was asymptomatic in both patients and resolved without therapy.

Our system’s maximum therapeutic field size is 15 cm in diameter on the clinical protocol. However, scalp angiosarcoma can potentially involve the whole scalp, and in such a case the entire scalp needs to be irradiated [15]. Our system can therefore only be used in the setting of localized disease. Even with this limitation, we expect that BNCT will become a promising treatment option for scalp angiosarcoma.

## Sources of support

This work was supported by a JSPS Grant-in-Aid for Young Scientists (Grant Number 19K17218) and a JSPS Grant-in-Aid for Young Scientists (B) (Grant Number 26860410), and partially supported by a JSPS Grant-in-Aid for Scientific Research (B) and (C) (Grant Number 19H03611 and 16K10410) and by the National Cancer Center Research and Development Fund (29-A-8), (26-A-18), and (23-A-46).

## Declaration of Competing Interest

The authors declare that they have no known competing financial interests or personal relationships that could have appeared to influence the work reported in this paper.
